# Dual localization of the carboxy-terminal tail of GLR3.3 in sieve element–companion cell complex

**DOI:** 10.1080/19420889.2023.2167558

**Published:** 2023-01-22

**Authors:** Qian Wu, Mengjiao Chen, Archana Kumari

**Affiliations:** a Shenzhen Branch, Guangdong Laboratory for Lingnan Modern Agriculture, Genome Analysis Laboratory of the Ministry of Agriculture, Agricultural Genomics Institute at Shenzhen, Chinese Academy of Agricultural Sciences, Shenzhen, Guangdong, China; b National Key Laboratory of Crop Genetics and Germplasm Enhancement, College of Horticulture, Nanjing Agricultural University, Nanjing, Jiangsu, China; c Plant Molecular Biology Unit, Division of Biochemical Sciences, CSIR-National Chemical Laboratory, Pune, India

**Keywords:** glutamate receptor, electrical signaling, wound signaling, sieve element, companion cell

## Abstract

Glutamate receptor-like (GLR) 3.3 and 3.6 proteins are required for mediating wound-induced leaf-to-leaf electrical signaling. In the previous study, we found that the carboxy-terminal tail of GLR3.3 contains key residues that are indispensable for its action in electrical signaling. In the present work, we generated plants that expressed the truncated C-tail fraction of GLR3.3. To our expectation, the truncated C-tail itself was not functional in propagating leaf-to-leaf signals. However, we identified that the C-tail-mVENUS fusion proteins had dual localization patterns in sieve elements and companion cells. In companion cells, the fusion proteins overlapped largely with the nucleus. We speculated that a possible nuclear localization signal is present in the C-tail of GLR3.3, paralleling the C-tails of the ionotropic glutamate receptors in animal cells. Our further findings on the C-tail of GLR3.3 open up new possibilities for the regulatory roles of the C-tails to GLR proteins.

## Introduction

Ionotropic glutamate receptor (iGluR) family proteins are widely studied in the central nervous system (CNS) of animals. iGluRs encode subunits of ligand-gated ion channels that mediate fast excitatory synaptic neurotransmission in mammals [1]. Plants are distinct from animals with regard to the fact that plants lack neron-like structures that allow fast excitatory reactions. However, iGluR homologues were identified in plants and were divided into three clades, with the clade 3 family proteins representing the most ancient members [2]. Since the last 20 y, a growing number of studies were carried out to uncover their biological roles [3]. Out of all the studies, members of clade 3 GLRs received great attention as they were found as genetic basis for wound-induced long distance electrical signaling and systemic defense responses [4], a process that parallels iGluR-mediated neurotransmission in animals. In Arabidopsis, loss-of-function mutations in two clade 3 *GLRs, GLR3.3* and *GLR3.6*, resulted in a failure in propagating leaf-to-leaf electrical signals (also termed as slow wave potentials, SWPs) that eventually dampened the defense activation in the undamaged region of the plants [4,5]. As ligand-gated ion channels, intensive structural, biochemical and physiological studies were performed to decipher their channel properties [6–8]. For extended knowledge of this part, we refer readers to some excellent reviews [9–11].

GLR proteins are large membrane-spanning proteins and have more than 900 amino acids. The cytosolic, soluble C-tails contain only less than 100 amino acids and are variable in sequences compared to other domains of the proteins. To understand the regulatory mechanisms of the GLR proteins in long-distance wound signaling pathway, in our recent work [12], we chose to study the carboxy-terminal tail (C-tail) of the GLRs, a fraction that was predicated to be cytoplasmic and has a potential to bind downstream signaling components. We identified the C-tail of GLR3.3 is required for long-distance wound signaling. Mutations in a trio of residues in the C-tail impaired its interaction with another protein called ISI1 and abolished GLR3.3 function in electrical signaling [12]. At present, these sites are the only characterized functional residues in GLR3.3. Clearly, the C-tails of GLRs are the least investigated, and their significance is underscored. More research needs to be done toward dissecting the regulatory roles of GLR C-tails in the signaling pathway.

## Materials and methods

### Plant materials

Arabidopsis Col-0 was used as wild type (WT) in this work. The T-DNA insertion line *glr3.3a* (SALK_099757) was reported in [4]. To generate transgenic plants expressing GLR3.3_promoter_: GLR3.3CT-mVENUS, GLR3.3CT (Fw: GGGGACAAGTTTGTACAAAAAAGCAGGCTTATGCAGATCATCCGTCAGCTCT, Rv: GGGGACCACTTTGTACAAGAAAGCTGGGTTGTCTAATGGATTTACCGAATT) was cloned into a Gateway entry vector L1-pDONR/Zeo-L2. Together with the *GLR3.3* promoters in the modified pUC57 containing L4/R1 overhangs [5] and mVENUS in the entry clone pEN-R2-mVENUS-L3, triple Gateway reaction was performed to recombine them into the destination vector pB7m34GW. *Agrobacterium* harboring the resulting construct was used to transform the *glr3.3a* mutant plants. T1 seeds were selected on half-strength MS medium with 40 μg/ml Basta. Independent T3 homozygous lines were used for the analysis in this study.

### Slow wave potential measurements

Leaf-to-leaf slow wave potentials (SWPs) were measured according to the descriptions in [4,5,13]. In all the cases, leaf 8 (L8) was the wounded and the SWPs were recorded from both L8 and the distal connected leaf 13 (L13).

### Confocal microscopy

To visualize the subcellular localization of the C-tail fusion protein, vein samples were prepared and cleared as described previously [12,14]. DAPI was used to stain the nuclei before imaging. The stained samples were visualized with a SP8 microscope (Leica Microsystems CMS GmbH, Mannheim, Germany). DAPI was excited at 408 nm and detected in a window of 430–470 nm. mVENUS was excited at 514 nm and detected in a range of 520–540 nm.

## Results and discussion

**Figure 1.**
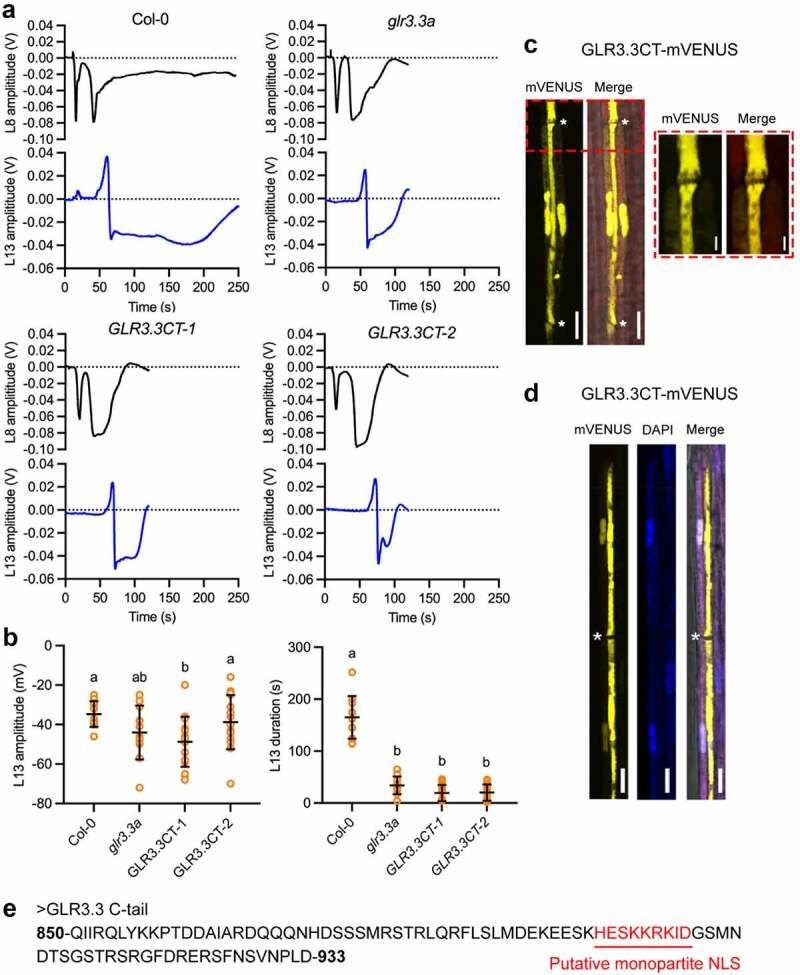
Functional and subcellular characterization of GLR3.3CT. (**a**) Representative traces of the SWPs in both L8 (black trace) and L13 (blue trace) from WT, *glr3.3a* mutants and two independent lines of transgenic plants expressing GLR3.3CT-mVENUS fusions (*GLR3.3CT-1* and *GLR3.3CT-2*). (**b**) Amplitude (upper panel) and duration (lower panel) quantification results of the L13 SWPs in different materials. Yellow circles represent individual recordings. *n* = 11–16. Data shown are mean ± SD. The different letters indicate significant differences after one-way ANOVA. (**c-d**) Subcellular localization of GLR3.3CT-mVENUS fusions. In (**c**), yellow is the signal from GLR3.3CT-mVENUS fusion protein. The junction between two sieve tubes was shown in the zoomed red box with dotted line. In (**d**), DAPI staining (blue) was further used to visualize the nuclei. Asterisks mark the positions of sieve plates. Bar = 2 μm in the enlarged image. Bar = 10 μm in all the other cases. (**e**) Amino acid sequence of GLR3.3 C-tail with a putative NLS highlighted by red. The NLS was predicted using cNLS Mapper [27].

In the course of deciphering the possible roles of GLR3.3 C-tail (GLR3.3CT), we also generated another set of transgenic plants that only expressed the truncated C-tail in fusion with a mVENUS tag in the *glr3.3a* mutant background. GLR3.3 without its C-tail failed to complement the mutant defect in propagating SWPs, indicating that C-tail is required for GLR3.3 function [12]. Moreover, the major pool of the GLR3.3 ΔCT protein localized properly as GLR3.3 WT protein in sieve elements (Figure 1d, 12). In this study, in comparison with the WT and *glr3.3a* mutants, plants only expressing GLR3.3 C-tail were also not capable of propagating leaf-to-leaf SWPs (*GLR3.3CT-1* and *GLR3.3CT-2*, Figure 1a-b). This is not surprising as a large proportion of proteins is defective in the transgenic lines. What is interesting to us is that GLR3.3CT-mVENUS localized dually in both the companion cells (CCs) and the sieve elements (SEs, Figure 1c-d). Notably, in the companion cells, mVENUS signals resided chiefly in the nuclei, whereas in the SEs, the signals were dispersed (Figure 1c-d). SE-CCs complexes are cytoplasmically connected via plasmodesmata-pore units (PPUs) that allow diffusion of molecules as large as 70 KDa [15]. Therefore, the detected signals in the SE-CC complex could be explained by the trafficking of the small mVENUS-tagged C-tail protein between SEs and CCs. However, in another study, when free GFP protein was expressed under CC-specific *SUC2* promoter, GFP fluorescence was spread in both phloem parenchyma cells and companion cells in Arabidopsis leaves [16]. This subcellular pattern is clearly different compared to GLR3.3CT-mVENUS in the companion cells, indicating that a nuclear localization signal (NLS) is likely present in the C-tail of GLR3.3. In consistence, a monopartite NLS was predicted within the C-tail of GLR3.3 (HESKKRKID, Figure 1e). This is also in line with our result in the recent work that GLR3.3CT interacted with an IMPA protein in yeast cells [12]. In 12, a C-tail variant carrying mutations in the above KKRK residues (mKKRK) showed similar localization pattern in SEs compared to the GLR3.3 WT protein. How mutations in KKRK or in the entire predicted NLS affect the C-tail localization remains to be investigated. Interestingly, in hyppocampal neurons, the cytoplasmic tail of the NR1-1a subunit of the NMDA receptor also interacts with importin α protein in an activity-dependent manner [17]. Further studies showed that the C-terminus of NR1-1a subunit could be cleaved by protease, and then translocated to neuronal nucleus, thereby regulating downstream signaling events [18]. This was proposed as an important mechanism mediating synapse-to-nucleus signaling, that eventually fine-tunes overall synaptic function [19]. Whether the potential nuclear localization signal in GLR3.3CT has a regulatory role toward GLR function remains an open question that requires in-depth exploration.

The intracellular tails of membrane proteins are direct targets in mediating signaling pathway. As another key player in wound-induced electrical signaling and defense responses [20], plasma membrane-localized H^+^-ATPase 1 (AHA1) protein consists of ten transmembrane segments, with the C- and N-terminal domains extending to cytosol [21,22]. The autoinhibitory C-termini domain is a key region in regulating H^+^-ATPase activity [23,24]. It was speculated that specific phosphorylation events on C-terminal domain of AHA1 contribute to the plant defense responses. In fact, molecular studies have shown that auto-inhibitory C-terminal regulatory domains of proton pumps are regulation targets in response to diverse environmental conditions that activate/inhibit proton pumping [25,26]. However, unlike GLRs, AHA C-tail truncation is expected to activate the pumps. Altogether, the C-tails of both GLRs and AHAs provide opportunities to characterize their mode of actions at the mechanistic level, and to reveal the downstream signaling cascade activating the defense pathway.
